# Phytochemical Characterization of Saudi Mint and Its Mediating Effect on the Production of Silver Nanoparticles and Its Antimicrobial and Antioxidant Activities

**DOI:** 10.3390/plants12112177

**Published:** 2023-05-30

**Authors:** Husam Qanash, Abdulrahman S. Bazaid, Naif K. Binsaleh, Bandar Alharbi, Nawaf Alshammari, Safa H. Qahl, Hayaa M. Alhuthali, Abdullatiff A. Bagher

**Affiliations:** 1 Department of Medical Laboratory Science, College of Applied Medical Sciences, University of Ha’il, Ha’il 55476, Saudi Arabia; ar.bazaid@uoh.edu.sa (A.S.B.); n.binsaleh@uoh.edu.sa (N.K.B.); b.alharbi@uoh.edu.sa (B.A.); 2 Department of Biological Sciences, University of Ha’il, Hail 81451, Saudi Arabia; naib.alshammari@uoh.edu.sa; 3 Department of Biology, College of Science, University of Jeddah, Jeddah 21589, Saudi Arabia; shqahal@uj.edu.sa; 4 Department of Clinical Laboratory Sciences, College of Applied Medical Sciences, Taif University, Taif 21944, Saudi Arabia; hmhuthali@tu.edu.sa; 5 Medical Laboratory, East Jeddah General Hospital, Jeddah 22253, Saudi Arabia; abagher@moh.gov.sa

**Keywords:** Saudi mint, plant natural product, silver nanoparticles (AgNPs), antimicrobial, antioxidant

## Abstract

The green synthesis of nanoparticles (NPs) is attracting enormous attention as a new area of study that encompasses the development and discovery of new agents for their utilization in different fields, such as pharmaceuticals and food. Nowadays, the use of plants, particularly medicinal plants, for the creation of NPs has emerged as a safe, ecofriendly, rapid, and simple approach. Therefore, the present study aimed to use the Saudi mint plant as a medicinal plant for the synthesis of silver nanoparticles (AgNPs) and to evaluate the antimicrobial and antioxidant activities of AgNPs compared to mint extract (ME). A phenolic and flavonoid analysis that was conducted by using HPLC indicated the presence of numerous compounds in the ME. Through an HPLC analysis, chlorogenic acid at a concentration of 7144.66 µg/mL was the main detected component in the ME, while catechin, gallic acid, naringenin, ellagic acid, rutin, daidzein, cinnamic acid, and hesperetin were identified in varying concentrations. AgNPs were synthesized by using ME and were confirmed via UV–visible spectroscopy at 412 nm of the maximum absorption. The mean diameter of the synthesized AgNPs was measured by TEM to be 17.77 nm. Spectra obtained by using energy-dispersive X-ray spectroscopy indicated that silver was the main element formation in the created AgNPs. The presence of various functional groups, analyzed by using Fourier transform infrared spectroscopy (FTIR), indicated that the mint extract was responsible for reducing Ag^+^ to Ag^0^. The spherical structure of the synthesized AgNPs was confirmed by X-ray diffraction (XRD). Furthermore, the ME showed reduced antimicrobial activity (a zone of inhibition of 30, 24, 27, 29, and 22 mm) compared with the synthesized AgNPs (a zone of inhibition of 33, 25, 30, 32, 32, and 27 mm) against *B. subtilis*, *E. faecalis*, *E. coli*, *P. vulgaris*, and *C. albicans*, respectively. The minimum inhibitory concentration of the AgNPs was lower than that of the ME for all of the tested micro-organisms, except for *P. vulgaris*. The MBC/MIC index suggested that the AgNPs revealed a higher bactericidal effect compared to the ME. The synthesized AgNPs exhibited antioxidant activity with a reduced IC_50_ (IC_50_ of 8.73 µg/mL) compared to that of the ME (IC_50_ of 13.42 µg/mL). These findings demonstrate that ME could be applied as a mediator for AgNPs synthesis and natural antimicrobial and antioxidant agents.

## 1. Introduction

Since ancient times, the *Mentha* species has been recognized as possessing several aromatic and pharmaceutical purposes, due to their potential antifungal, antioxidant, and inhibitory action regarding mycotoxin production in addition to their potential use as natural food preservatives [1]. A diversity of secondary metabolites was associated with the genus of *Mentha*, as it was applied in traditional medicines, household remedies for digestive disorders and food additives, and was utilized to treat inflammation of the mouth; minimize bacterial infection; and also as a tonic, mild laxative, culinary herb, and astringent [2]. These biochemical features are principally caused by the presence of various aromatic and phenolic chemicals in the various sections of the *Mentha* spp. The chemical composition of the *Mentha* species extracts may depend on the specific growing season, species, and climatic conditions [3,4]. For the present investigation, two biological potentials, including the antimicrobial and antioxidant activities of mint plant extract and AgNPs, were studied because using the mediated plant for AgNPs synthesis is more efficient in the presence of safe compounds, which play a vital role as antioxidants. This study also focused on antimicrobial activities because AgNPs can be used to fight numerous micro-organisms and therefore may be used as a topical ointment to repress human pathogenic micro-organisms.

Several cultivated *Mentha* species were studied in Saudi Arabia, illustrating their potential use as a natural antioxidant and antifungal treatment [5,6,7]. Leaves of cultivated *M. longifolia*, collected from the Al Baha region, were investigated and chemically analyzed to detect the active constituents that combat pathogenic micro-organisms [8]. *M. longifolia*, *M. microphylla*, and *M. australis* were planted in Al Madinah Al Munawwarah, Saudi Arabia. Their analysis showed they were able to provide different amounts of essential oils [9]. Aldogman et. al. [5] recently reported that *M. suaveolens* L. in Saudi Arabia could be used as a natural flavoring, antioxidant, and antifungal agent. They reported that the extract of *M. suaveolens* L. displayed the highest antifungal activity (53%) and more than 90% antioxidant activity [5]. The antioxidant activity of two Saudi *Mentha* species was evaluated by Ahmed et. al. [6], who found that *Mentha longifolia* exhibited a stronger antioxidant than *M. pulegium*, which was attributed to its high flavonoid content. In addition, Anwar et. al. [7] reported that several biological activities, such as serving as an antioxidant and antimicrobial, were due to the presence of certain phenolic compounds. In another report, *M. suaveolens* L. was used as a strong inhibitor of fungal growth as well as mycotoxins production, and also reflected antioxidant activities [1]. *Candida albicans* and *Aspergillus niger* were inhibited by the volatile oils of *M. suaveolens* [10]. Several biological activities were associated with *M. suaveolens* volatile oils, particularly antioxidant activity [11]. Farnad et. al. [12] reported that the most widely detected phenolic acids were caffeic acid, followed by cinnamic acid, protocatechuic acid, gentisic acid, vanillic acid, and hydroxybenzoic acid. In addition, the antioxidant activity of three species, including *M. pulegium*, *M. spicata*, and *M. suaveolens*, was recently documented due to the existence of phenolic compounds [13].

Several new discoveries in the fields of physics, engineering, biology, and agriculture have been documented and developed in the last decade due to nanotechnology [14]. Various methods, including chemical, physical, physicochemical, and biological approaches, were applied for the purpose of nanoparticles (NPs) synthesis, but the biological method, using bacteria, fungi, algae, and plants, has been characterized as the main safe, ecofriendly, and cheap process for NPs creation [15,16,17,18]. During the last decade, many scientists have investigated the use of various plant extracts in the fabrication of silver nanoparticles (AgNPs). However, due to their availability and widespread use, common medicinal and edible plants have frequently been the subject of important research studies [19]. As several previous studies mention, plants are rich in various secondary metabolites [20,21,22,23] that play an essential role in the reduction and capping process throughout the creation of NPs [19]. One of the main constituents (menthol) of *Mentha piperita* is responsible for the creation of AgNPs [24]. The creation of AgNPs by *Mentha arvensis* [25] and *Mentha pulegium* [26] were reported. Several biological utilities of AgNPs, particularly green ones, such as being antimicrobial, antioxidant, anticancer, antimalarial, and antidiabetic, were documented [27]. In addition, AgNPs showed an inhibition of phytopathogenic fungi, such as *Fusarium solani* and *Alternaria alternata* [26]. The inhibitory action of AgNPs was also reported against multidrug-resistant bacteria, such as *Methicillin-resistant S. aureus*, *Salmonella enterica*, *E. coli*, and *P. aeruginosa* [27,28,29]. In other studies, the antimicrobial activity against bacteria and fungi as well as mycotoxins was enhanced when the plant extract incorporated AgNPs [30,31]. Several mechanisms of the antimicrobial activities of AgNPs were explained through the interactions with DNA and cytoplasmic contents of microbial cells, leading to the blocking of cell division [25]. The ecofriendly, cost-effective creation of AgNPs is being requested by both the public and environmentalists for pharmacological and ecological applications. The current study suggests that the creation of AgNPs via the leaf extract of *M. longifolia* can help to overcome the pathogenic microbes. Additionally, studying the antioxidant activity of synthesized AgNPs can facilitate the comparison of the antioxidant activity between the mint extract and the created AgNPs. An HPLC analysis of the mint extract was also conducted in addition to exploring its antimicrobial and antioxidant properties.

## 2. Results and Discussion

### 2.1. Phenolic and Flavonoid Characterizations of Mint Extract (ME)

**Figure 1:**
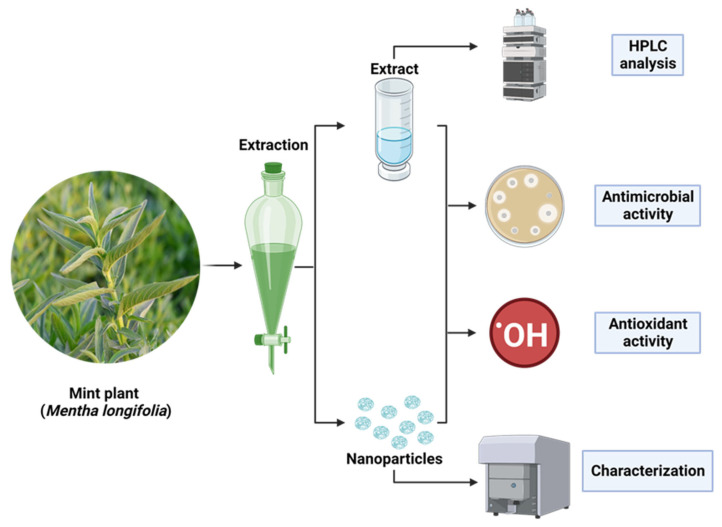
Image illustrating the mint plant (*Mentha longifolia*) and mint mediate AgNPs synthesis, which was subjected to effective extraction methods and several appropriate assays to identify the phytochemical analysis of the mint extract and the antimicrobial and antioxidant activities of the extracted product compared to the synthesized AgNPs.

**Figure 2:**
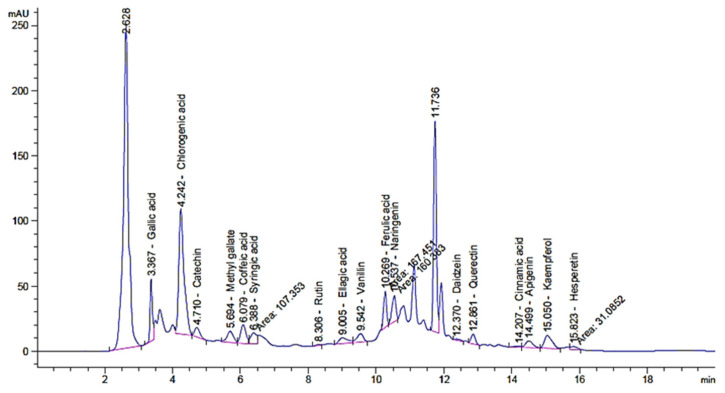
High-performance liquid chromatography (HPLC) of the detected phenolic and flavonoid constituents in mint extract.

Saudi mint was selected for the current study due to its traditional and medicinal applications. The antimicrobial and antioxidant activities of the extract as well as the created AgNPs were evaluated. The experiments on mint and mint-mediated AgNPs synthesis were described (Figure 1). The phenolic and flavonoid constituents of Saudi mint were analyzed via HPLC. The analysis showed the existence of various constituents with a dissimilar retention time, area, area%, and levels of concentration (Table 1 and Figure 2). In addition, the whole chemical construction of the detected compounds was documented (Figure S1). It was clear that the chlorogenic acid was found in a very high concentration (7144.66 µg/mL) compared to the other identified compounds. Catechin, gallic acid, and naringenin were detected in a narrow concentration range of 966.93, 980.13, and 958.50 µg/mL, respectively, followed by ellagic acid (841.23 µg/mL). Hesperetin (83.77 µg/mL), rutin (47.82 µg/mL), daidzein (43.25 µg/mL), and cinnamic acid (10.40 µg/mL) were detected at low concentrations. Chlorogenic acid, according to the study conducted by Yan et. al., showed an effective improvement in tolerance to glucose as well as insulin resistance and could decrease the abundance of microbial infections [32]. Choi et. al. reported that rutin could minimize the severity of inflammation and blood coagulation [33]. Several studies focused on the pharmacological applications of quercetin, as it could be used to prevent cancer proliferation, protect against Alzheimer’s Disease, and inhibit diabetes development, as well as possessing some antioxidant properties [34,35]. In another previous study, both catechin and gallic acid exhibited retardation potential against *Helicobacter pylori*, but a higher inhibitory effect was attributed to gallic acid compared to catechin [36]. Moreover, Bai et. al. documented that gallic acid had an anti-inflammatory action [37]. Ellagic acid also displayed several pharmaceutical activities, including antioxidant, antiproliferative, anti-inflammatory, antimutagenic, antiallergic, antiatherosclerotic, antiallergic, neuroprotective, and nephroprotective properties [38]. In the current study, the detected flavonoids (kaempferol and quercetin) were reported to acquire antifungal and antibacterial properties, and the results were consistent with Jan et. al. [39].

### 2.2. Characterization of Phyto-Created AgNPs by Mint Plant (ME)

**Figure 3:**
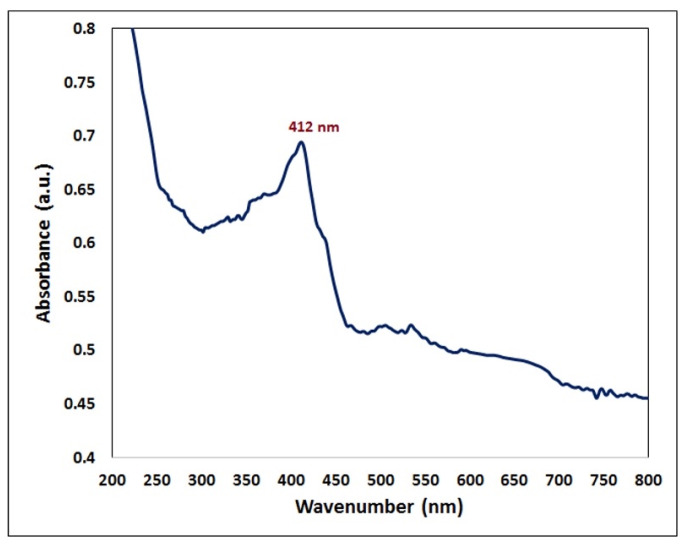
UV–visible spectra of the phyto-created AgNPs.

**Figure 4:**
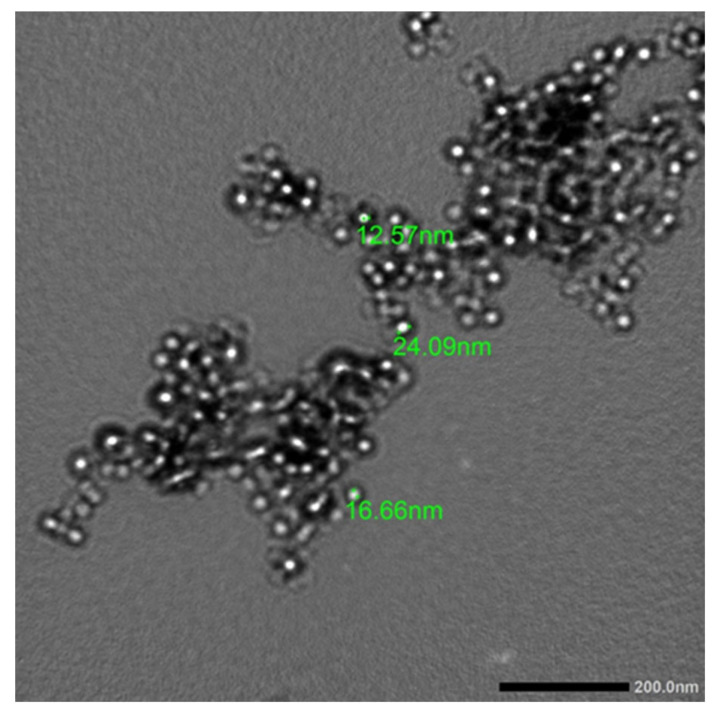
Transmission electron microscopy (TEM) of phyto-created AgNPs.

In this study, the production of AgNPs in the mixture with a surface plasmon resonance (SRP) spectrum of 412 nm of maximum absorption was validated by using a UV–visible optical microscope (Figure 3). The obtained results were analogous to those published in the literature, in which biological extract was employed to produce AgNPs, and the UV–visible absorbance spectra indicated that the SPR band for Ag particles was in the 400–450 nm range [40,41,42]. The creation of AgNPs was reported when using several *Mentha* spp., such as *Mentha arvensis* and *Mentha pulegium* [25,26]. The synthesized AgNPs were characterized via transmission electron microscopy (TEM) in order to detect the morphology and diameter of the NPs (Figure 4). The synthesized AgNPs tended to be spherical with a mean diameter of 17.77 nm. The findings of the current study were consistent with Rizwana and Alwhibi [26], where the size of the created AgNPs, which were created by using *M. pulegium* extract, ranged between 4 and 60 nm. Moreover, the results were consistent with Bashir et. al. [40], who found that the size of the AgNPs created by using *M. asiatica* was 29 nm.

**Figure 5:**
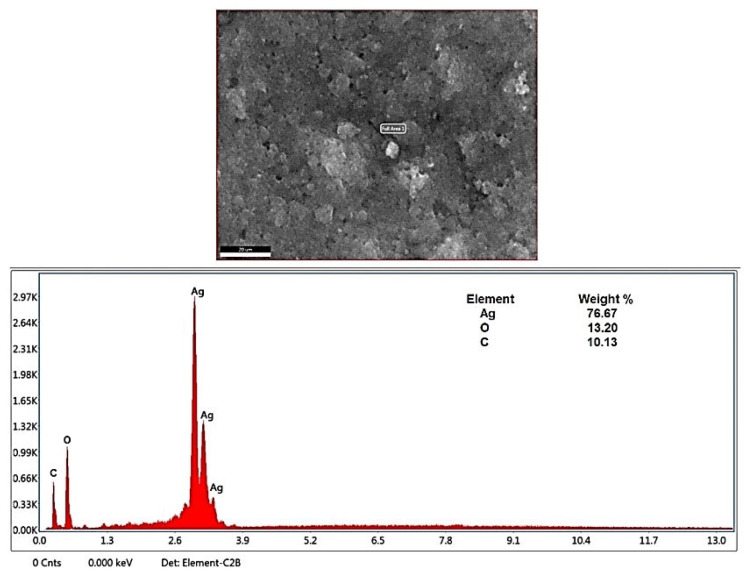
SEM-EDX analysis for phyto-created Ag-NPs.

The quantitative elemental compositions of phyto-created AgNPs were analyzed and compared by using a scanning electron microscopy–energy dispersive X-ray (SEM-EDX) analysis. The existence of spherical AgNPs was found, and an elemental analysis confirmed the fabrication of AgNPs at a rate of 76.67% (Figure 5). The EDX spectra also clearly showed the presence of AgNPs with a strong peak at 3 KeV, which indicated that silver was the major element formation. Moreover, the distinct peak signals of oxygen and carbon were recorded to be 13.20% and 10.13%, respectively, which was related to the presence of metabolites, such as carbohydrates, proteins, and others [43,44].

**Figure 6:**
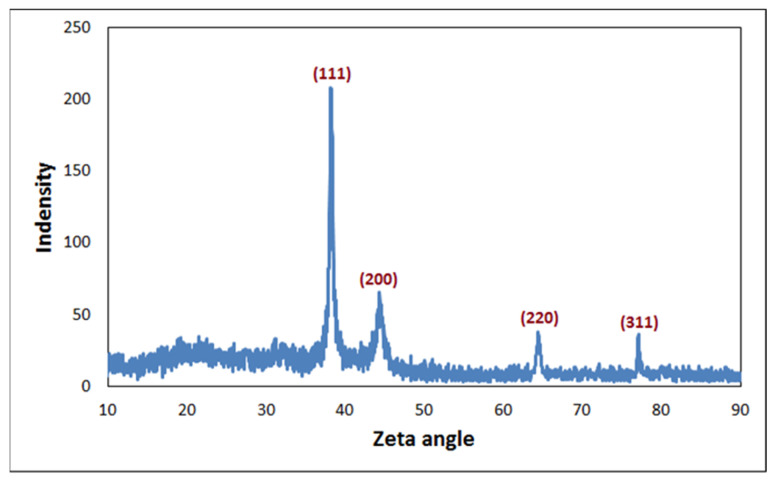
X-ray diffraction (XRD) analysis for phyto-created AgNPs.

An XRD pattern of the AgNPs that demonstrated the spherical structure of the formed AgNPs was observed (Figure 6). The typical diffraction peaks, at 2θ = 38.2°, 44.3°, 64.4°, and 77.1°, were ascribed to (111), (200), (220), and (311), respectively, which reflected the planes of the silver cubic face of the cubic-centered (FCC) crystalline form (JCPDS No 87-0720). The results showed that the AgNPs biosynthesized by reducing Ag^+^ ions from the biological extracts had a crystalline nature, and the sharp peak at 2θ of ~38.2° indicated the position (111) of Ag that corresponded to the face cubic center [43,45]. The results indicated that the biosynthesized AgNPs were composed of crystalline silver nano with a high purity. 

**Figure 7:**
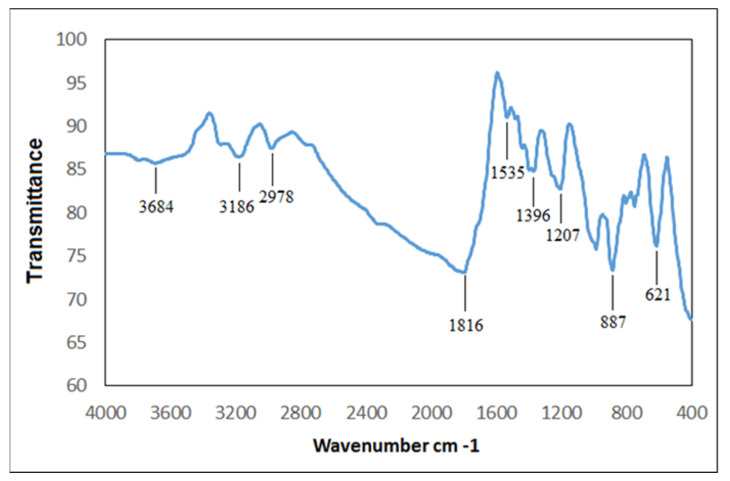
Fourier-transform infrared spectrum of phyto-created AgNPs.

**Figure 8:**
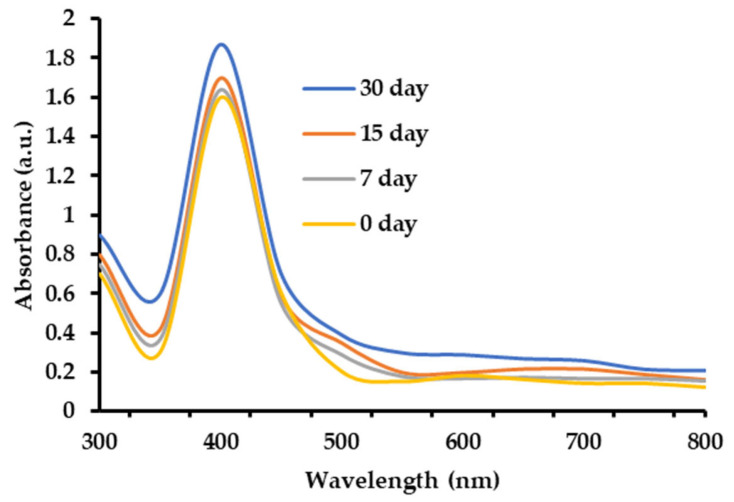
The UV/Vis spectra of AgNPs at different days at 30 °C.

The functional groups found in the AgNPs were characterized by using Fourier-transform infrared (FTIR) analysis [40,46]. The FTIR of the biogenic AgNPs displayed varying peaks at 3684, 3186, and 2978 cm^−1^ (OH, NH stretching, and aliphatic primary amines and OH broad stretching, respectively) (Figure 7). Other peaks appeared at 1816 cm^−1^ and 1535 cm^−1^ (amide-I band) and 1396 and 1207 cm^−1^ (CN stretching of the amines). Interestingly, the FTIR peaks at 887 and 621 cm^−1^ corresponded to the formation of Ag. Moreover, the stability of the AgNPs under different storage conditions at 30 °C was documented through UV/Vis spectra (Figure 8). The surface plasmon resonance (SPR) bands were symmetrical, where the peaks’ position remained stable over time for up to 30 days, which indicated that the AgNPs were stable.

### 2.3. Antimicrobial Activity of Mint Extract and Phyto-Created AgNPs

**Figure 9:**
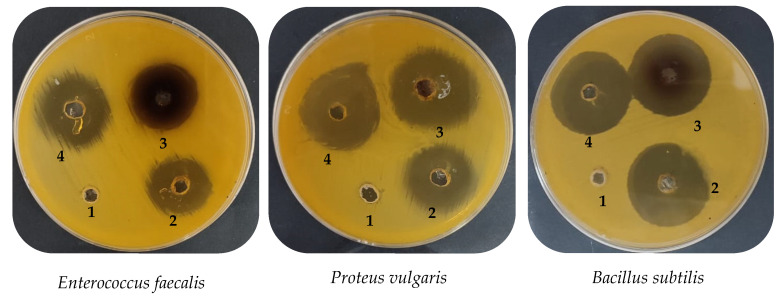

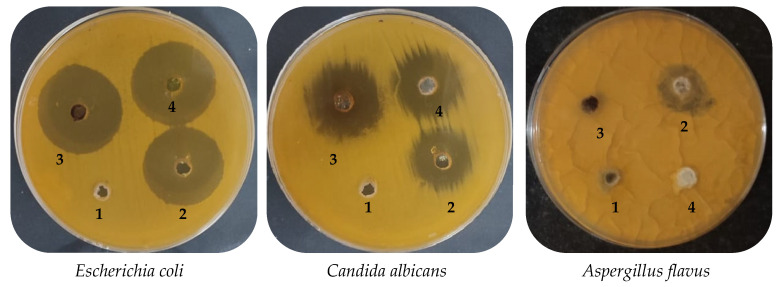
Antimicrobial activity of mint extract and created AgNPs; negative control (1); positive control (2); AgNPs (3); and mint extract (4).

The mint extract (ME) exhibited antimicrobial activity against all the tested microbes, except for *A. flavus*, with various inhibition zones of 30.67, 24.33, 27.83, 29.67, and 22.83 mm (Table 2 and Figure 9); various MICs of 15.70, 15.77, 7.87, 7.90, and 62.67 µg/mL; and various MBCs of 31.83, 62.33, 15.50, 15.67, and 124.33 µg/mL, compared with the inhibition zones of positive control gentamycin/ketoconazole against *B. subtilis*, *E. faecalis*, *E. coli*, *P. vulgaris*, and *C. albicans*, respectively. Various bacterial species, including *Pseudomonas aeruginosa*, *Escherichia coli*, *Staphylococcus aureus*, *Shigella flexneri*, and *Klebsiella pneumoniae* were inhibited by using an ethanolic extract of *M. arvensis*, and *S. aureus* was the most susceptible (a 21 mm inhibition zone diameter) of all of the tested bacteria in a similar way to standard antibiotic tetracycline [47]. Moldovan et. al. documented the antimicrobial activity of five *Mentha* spp., such as *M. suaveolens* var. *rispate*, *M. rotundifolia*, *M. spicata* subsp. *rispate*, *M. piperita* var. *officinalis F. pallescens*, and *F. rubescens*, with inhibition zones ranging from 8 to 26 mm against *S. aureus*, *Listeria monocytogenes*, *Salmonella typhimurium*, *E. coli*, and *C. albicans* [48]. 

The phyto-mediated AgNPs that were created by using mint extract (ME) exhibited greater antimicrobial activity compared to the ME. Moreover, the AgNPs showed a promising inhibition zone, compared to standard antibiotics tested against bacteria and *C. albicans*, with the exception of *E. coli* (Table 2 and Figure 9). These results are promising, in comparison with Aziz and Jassim [49], where the diameters of the inhibition zones of the AgNPs synthesized with mint against *B. subtilis* and *E. coli* were 25 mm and 20 mm, respectively. In another previous study, the ability of AgNPs synthesized by *M. longifolia* to inhibit the phytopathogens bacteria was recorded against *Pectobacterium carotovorum, Xanthomonas vesicatoria*, *Xanthomonas oryzae*, and *Ralstonia solanacearum*, and potential inhibitory activity was detected between 2–12 μg ml^−m^ [50].

The minimum inhibitory concentration (MIC) of AgNPs was reported to be lower in the case of *E. faecalis* (15.53 µg/mL), *E. coli* (7.73 µg/mL), and *C. albicans* (31.33 µg/mL), but higher (7.83 and 15.57 µg/mL) in the case of *B. Subtilis* (7.83 µg/mL) and *P. vulgaris* (15.57 µg/mL) compared to the ME (Table 2). In addition, the antimicrobial property of the synthesized AgNPs was evaluated against common oral microbes, including *Streptococcus mutans*, *Staphylococcus aureus*, *Candida albicans*, and *Enterococcus faecalis* [51]. AgNPs, synthesized by using the leaves of Saudi *Mentha pulegium*, displayed antibacterial activity against *Listeria monocytogenes*, *Salmonella typhimurium*, *Staphylococcus aureus*, *Pseudomonas aeruginosa*, *Escherichia coli*, and *Bacillus cereus*, with the diameters of the inhibition zones ranging from 17 to 26 mm. The most effective antimicrobial activity was reported against *E. coli*, while less effective antimicrobial activity was observed against *L. monocytogenes* [52]. AgNPs were found to be more effective than commercial antibiotics in the current study, in line with Naghmouchi et. al. [52]. The cidal effect of the extract or AgNPs was determined by calculating the MBC/MIC Index, where the cidal properties increased as the index of the MBC/MIC decreased. Values less than four indicated that the present ME and AgNPs exerted a cidal effect. The mechanisms of the antimicrobial activity of phyto-mediated AgNPs were studied in several reports, where the denaturation of bacterial protein [53], disorders of the bacterial respiratory chain [19], and lipopolysaccharides changes [54] were reported.

### 2.4. Antioxidant Activity of Mint Extract and Phyto-Created AgNPs

The rise in DPPH scavenging (%) indicated the efficient antioxidant activity of the ME and AgNPs. There was a relationship between antioxidant activity and the concentration of either the ME or AgNPs (Table 3). A promising IC_50_ was recorded when using ME (13.42 µg/mL); however, the AgNPs were more effective with a lower IC_50_ (8.73 µg/mL). In the present study, the antioxidant activity of the ME and AgNPs was compared to that of ascorbic acid, which differed slightly, particularly at high concentrations of 500 and 1000 µg/mL. It was common to record a low value for IC_50_ (8.73 µg/mL) when using ascorbic acid, but the IC_50_ values of the ME and AgNPs were surprisingly low. The most frequently detected flavonoid in the ME was reported to execute several biofunctional antioxidant activities. The findings were consistent with previous studies based in Saudi Arabia, where *M. longifolia* and *M. pulegium* displayed efficient antioxidant activity but with different levels of activity [6]. Additionally, the ethanolic extract of fresh or dry *M. suaveolens* exhibited a strong antioxidant potential [5]. Mint extract (ME) appeared to represent a promising antioxidant, due to its enriched content of rutin (252.16 µg/g) [54] and naringenin (53.22 µg/g) [55] in a whole plant. Therefore, the AgNPs that were synthesized by using *ME* could be applied to the treatment of several diseases that are affected by oxidative stress. The findings of the current study are in line with a recent study conducted by Bashir et. al., where the green fabricated AgNPs, that were created by utilizing leaf extract of *M. asiatica*, displayed a significant antioxidant potential [40]. Due to the several side effects caused by a synthetic antioxidant, researchers may consider using a natural antioxidant as a safe alternative [56].

## 3. Materials and Methods

### 3.1. Chemicals Used in the Study

All of the materials used in the current study were obtained from Sigma Aldrich, (Taufkirchen, Germany), in an analytical grade form.

### 3.2. Collection and Extraction of the Mint Plant

Fresh mint plants (*Mentha longifolia*) were collected from local markets in the cities of Medina and Hail, Saudi Arabia. Areal parts, including the leaves and stems, were washed in distilled water, and then dried at 30 °C until a constant weight was obtained. The dried collected plant (100 g) was ground down and immersed in 300 mL of methanol under a shaking condition by using a magnetic stirrer for 24 h. The mixture was centrifuged for 10 min at 5000 rpm. The obtained supernatant was concentrated by using a rotary evaporator before being completely concentrated and redissolved in DEMSO, and it was stored at 4 °C for further study.

### 3.3. Detection of the Phenolic and Flavonoid Constituents of Mint Extract via HPLC 

A total of 5 μL of mint extract as well as the AgNPs were added to the HPLC device (Agilent 1260 series, Agilent Technologies, Santa Clara, CA, USA) once the extraction process had been completed. The Eclipse C18 column employed had the following specifications: 4.6 mm and 250 mm, i.e., 5 μm and 40 °C. During the mobile phase, two buffers were used: buffer A (military Q water and 0.05% trifluoroacetic acid) and buffer B (acetonitrile and 0.05% trifluoroacetic acid). A flow rate of 0.9 mL/min was used to apply the buffers. The serial dilutions for buffer A were 82, 80, 60, 60, 82, 82, and 82% for 0, 0–5, 5–8, 12–15, 15–16, and 20–20 min, respectively. A flow rate of 0.9 mL/min was used for the buffers. The mobile phase took 20 min to run, with the following serial dilutions of buffer A: 82, 80, 60, 60, 82, 82, and 82% for 0, 0–5, 5–8, 12–15, 15–16, and 20–20 min, respectively. The presence of phenolic compounds and flavonoids was determined by using an ultraviolet (UV) detector with a wavelength of 280 nm. Based on the injected standard compounds, the amount of each was recorded [16,57].

### 3.4. Synthesis of Silver Nanoparticles (AgNPs) 

The aqueous solution of 1 mM of AgNO_3_ was prepared by using distilled water. A total of 10 mL of mint extract was added to 90 mL of the aqueous solution of AgNO_3_. The reaction mixture was kept at 70 °C for 40 min, and the color change was subsequently observed. A color change from yellow to brown was used to detect the creation of AgNPs via Ag ion reduction.

### 3.5. Characterization of Synthesized AgNPs

The reaction mixture containing the AgNPs was characterized by using ultraviolet–visible spectroscopy (Perkin Elmer, MA, USA) to measure the absorption spectrum in a range from 200 up to 800 nm. The morphology and size of the created AgNPs were characterized by using a transmission electron microscope (TEM) (JEOL-JEM-Plus-1400, Tokyo, Japan). Fourier-transform infrared (FTIR) spectroscopy was applied to characterize the functional groups that are responsible for encapsulating and reducing the Ag ions to AgNPs by utilizing a pellet of potassium bromide on a Thermo Fisher Spectrometer (Thermo Scientific Model-Nicolet 6700, Hillsboro, OR, USA). The infrared spectrum was measured at a range of wavelength of 400–4000 cm^−1^. The elemental contents of the synthesized AgNPs were examined with an energy dispersive X-ray detector (EDX-FESEM-model no-JSM-7610F, Akishima, Japan) at an accelerating 30 kilo voltage. An X-ray diffraction (XRD) (Model diffractometer, Shimadzu 7000, Tokyo, Japan) analysis was conducted to determine the crystal structure of the created AgNPs.

### 3.6. Antimicrobial Activity of Mint Extract and AgNPs Using Agar Diffusion Method

All of the tested micro-organisms were provided by the American Type Culture Collection (Manassas, VA, USA). Mint extract and the AgNPs’ ability to inhibit micro-organisms (*Bacillus Subtilis* (ATCC 6633), *Enterococcus faecalis* (ATCC 10541), *Escherichia coli* (ATCC 8739), *P. vulgaris*, *Candida albicans* (ATCC 10221), and *Aspergillus flavus*) was evaluated in accordance with the Clinical and Laboratory Standards Institute (CLSI) guidelines. An agar diffusion technique was used to test the antibacterial activity. Mueller–Hinton agar (MHA) plates were used to maintain the tested bacteria and *C. albicans*, while a Dox medium was used for the tested fungus. A seedling of 2 × 10^8^ CFU/mL of tested bacteria and 2 × 10^6^ of the tested fungus was applied, and the MHA and Dox agar media were inoculated. A 6 mm well was then made in the inoculated media, and 100 µL of the mint extract and AgNPs was separately added to the wells. The cultured plates underwent a 24-h incubation period at 37 °C for the bacteria and *C. albicans*, while the tested fungi were incubated at 30 °C for 3 days. The inhibition zone that appeared following the incubation period was measured. The solvent employed for the extraction was applied as a negative control, while the standard antibiotic/antifungal treatments (Gentamycin, 0.1 mg/mL and ketoconazole, 0.1 mg/mL) were applied to inhibit the bacteria and fungi, respectively; additionally, silver nitrate (200 µg/mL) was utilized as a positive control [58,59]. 

### 3.7. The Minimal Inhibitory Concentration (MIC) Experiment

To determine the MIC of the mint extract and AgNPs against the tested micro-organisms, a Mueller–Hinton broth was used as the microdilution broth [60]. Various serial dilutions containing concentrations of mint extract and AgNPs ranging from 0.98 to 1000 μg/mL were prepared. We used 96-well polystyrene microtitrate plates, with 200 μL of each appropriate dilution of mint extract and AgNPs being dispensed per well. A fresh-tested micro-organism culture inoculum was created in sterile NaCl (0.85%) to match the required 1.0 McFarland turbidity. A final concentration of 2 × 10^6^ CFU/mL was obtained by inoculating each well with a total of 2 mL of the tested micro-organisms, and the plates were incubated at 35 °C for three days. The minimal inhibitory concentration was visually assessed to determine the lowest concentration required to inhibit the tested micro-organisms. Each microplate contained an inoculum of the tested micro-organisms without either mint extract or AgNPs as a positive control, and the extract and AgNPs without the tested micro-organisms as a negative control [61].

### 3.8. The Minimal Bactericidal Concentration (MBC) Experiment

A total of 100 mL of the tested micro-organisms that showed no growth onto the sterile Mueller–Hinton agar plates were subcultured from each well [62]. The cultivated plates were incubated at 35 °C for three days. The MBCs were detected as the lowest concentration of mint extract and AgNPs that visually showed no growth for the tested micro-organisms. To determine whether the mint extract and AgNPs possessed bactericidal or bacteriostatic properties, the ratios of the MBC/MIC were calculated. The extract was considered to be an efficient bactericidal agent if the MBC/MIC ratio was less than four times the MIC [63].

### 3.9. The Antioxidant Activity of the Mint Extract and AgNPs 

Mint extract’s capacity to scavenge free radicals was assessed by using a DPPH (1,1-diphenyl-2-picrylhydrazyl) assay. A 0.1 mM DPPH solution in ethanol was created. In total, 3 mL of mint extract and AgNPs at different concentrations (ranging from 1.95 to 1000 µg/mL) were mixed with 1 mL of a 0.1 mM DPPH solution in ethanol. Only ethanol-soluble extracts were used, and various concentrations of those extracts were created by diluting the original extract. The mixture was shaken vigorously and then left to stand at 25 °C for 30 min. In the following step, a UV-VIS Milton Roy spectrophotometer was used to measure the absorbance at 517 nm. Ascorbic acid was used as a standard antioxidant agent. The concentration of mint extract or AgNPs necessary to inhibit 50% of the DPPH free radical (IC_50_) was calculated by utilizing a Log dose of the inhibition curve, as described by Al-Rajhi et. al. [23]. The percentage of the DPPH scavenging influence was recorded via the following formula:

$$\mathrm{DPPH}\;\mathrm{scavenging}\;\left(\%\right)=\frac{A_{0}-A_{1}}{A_{0}\;}\times 100$$

where the control reaction absorbance is A_0_, and the mint or AgNPs’ reaction absorbance is A_1_.

### 3.10. Statistical Analysis

The biological activities of the mint extract and AgNPs were studied in triplicate, and the results were assessed as a mean value of the standard deviation (±SD).

## 4. Conclusions

In the current investigation, Saudi mint extract appeared to represent an ecofriendly source for creating AgNPs. In addition, several characterizations, including UV–visible spectroscopy, TEM, FTIR, and XRD confirmed the complete synthesis of AgNPs. The mint extract displayed numerous phenolic and flavonoid compounds, which were identified by HPLC, such as catechin, gallic acid, ellagic acid, naringenin, ferulic acid, kaempferol, and Quercetin. The Saudi mint extract was also shown to be enriched with chlorogenic acid. The findings of the present study indicate that mint extract and the synthesized AgNPs possess significant antimicrobial potential to combat several bacteria, including *Bacillus Subtilis* (ATCC 6633), *Enterococcus faecalis* (ATCC 10541), *Escherichia coli* (ATCC 8739), *P. vulgaris*, and unicellular fungus (*C. albicans*). Additionally, promising antioxidant activities were attributed to mint extract and AgNPs with an IC_50_ of 13.42 µg/mL and 8.73 µg, respectively, compared to the IC_50_ value of ascorbic acid (3.45 µg/mL). However, the AgNPs displayed the highest level of antimicrobial and antioxidant activities, which supported the use of nanotechnology to permit and enhance the application of plant-derived products during treatment.

## Figures and Tables

**Table 1:** Identified contents of phenolic and flavonoid in mint extract.

Compound	Retention Time	Area	Area (%)	Concentration (µg/mL)
Gallic acid	3.367	221.00	3.60	966.93
Chlorogenic acid	4.242	1031.15	16.81	7144.66
Catechin	4.710	78.59	1.28	980.13
Methyl gallate	5.694	114.42	1.87	316.86
Caffeic acid	6.079	160.35	2.61	631.52
Syringic acid	6.388	107.35	1.75	379.83
Rutin	8.306	8.03	0.13	47.82
Ellagic acid	9.005	81.01	1.32	841.23
Vanillin	9.542	85.37	1.39	189.64
Ferulic acid	10.269	167.45	2.73	574.81
Naringenin	10.537	160.38	2.62	958.50
Daidzein	12.370	13.93	0.23	43.25
Quercetin	12.861	64.90	1.06	428.51
Cinnamic acid	14.207	11.1515	0.18	10.40
Apigenin	14.499	74.85	1.22	283.88
Kaempferol	15.050	156.56	2.55	570.72
Hesperetin	15.823	31.09	0.51	83.77

**Table 2:** The inhibitory activity, minimum inhibitory concentration (MIC), and minimal bactericidal concentration (MBC) of mint extract (ME) and synthesized AgNPs against the tested microbes.

Tested Organism	Mean Inhibition Zones (mm)			MIC (µg/mL)		MBC (µg/mL)		MBC/MIC Index		Mean Inhibition Zones (mm) of AgNO_3_
	AgNPs	ME	Control *	AgNPs	ME	AgNPs	ME	AgNPs	ME	200 µg/mL
*Bacillus subtilis*	33.67 ± 1.15	30.67 ± 0.58	30.17 ± 1.26	7.83 ± 0.06	15.70 ± 0.17	31.17 ± 0.14	31.83 ± 1.01	3.98	2.03	24.22 ± 1.15
*Enterococcus faecalis*	25.33 ± 1.53	24.33 ± 0.58	23.17 ± 0.29	15.53 ± 0.12	15.77 ± 0.15	31.37 ± 0.20	62.33 ± 0.29	2.02	3.95	14.33 ± 1.53
*Escherichia coli*	30.00 ± 1.73	27.83 ± 0.29	30.33 ± 0.29	7.73 ± 0.12	7.87 ± 0.06	7.90 ± 0.17	15.50 ± 0.17	1.02	1.97	21. 33 ± 1.53
*Proteus vulgaris*	32.33 ± 1.53	29.67 ± 0.58	26.00 ± 0.50	15.57 ± 0.06	7.90 ± 0.10	15.70 ± 0.17	15.67 ± 0.12	1.01	1.98	20.33 ± 1.15
*Candida albicans*	27.50 ± 0.87	22.83 ± 0.29	22.17 ± 0.29	31.33 ± 0.14	62.67 ± 0.29	63.0 ± 0.87	124.33 ± 1.15	0.02	1.98	16.00 ± 1.00
*Aspergillus flavus*	NA	NA	17.33 ± 0.58	-	-	-	-	-	-	NA

* Gentamycin and ketoconazole were applied as positive controls for bacteria and fungi, respectively.

**Table 3:** DPPH scavenging (%) of mint extract (ME) and ascorbic acid.

Concentration (µg/mL)	Ascorbic Acid		ME		AgNPs	
	DPPH Scavenging (%)	SD	DPPH Scavenging (%)	SD	DPPH Scavenging (%)	SD
1000	95.5	0.002	92.1	0.002	93.7	0.003
500	92.9	0.004	85.6	0.002	88.6	0.002
250	91.1	0.005	79.3	0.002	83.0	0.003
125	84.9	0.006	72.6	0.004	76.3	0.001
62.50	76.4	0.006	65.7	0.004	69.9	0.003
31.25	69.6	0.004	59.5	0.001	63.2	0.005
15.63	62.6	0.005	52.1	0.007	56.1	0.006
7.81	54.7	0.003	45.5	0.003	48.5	0.004
3.90	44.3	0.002	35.8	0.006	41.6	0.005
1.95	40.2	0.007	30.5	0.004	35.0	0.011
0	0.0	0.004	0.0	0.016	0.0	0.016
IC_50_	3.45 µg/mL		13.42 µg/mL		8.73 µg/mL	

## Data Availability

All data are available within the manuscript.

## Supplementary Materials

The following supporting information can be downloaded at: https://www.mdpi.com/article/10.3390/plants12112177/s1, Figure S1: Chemical constructions of the identified phenolic and flavonoid in mint extract.
