# Supplementary material for: Phytochemical Characterization of Saudi Mint and Its Mediating Effect on the Production of Silver Nanoparticles and Its Antimicrobial and Antioxidant Activities
Source: Plants (Basel). 2023 May 30;12(11):2177. doi: 10.3390/plants12112177 (PMC10255414; doi:10.3390/plants12112177)
Supplement: Supplementary file 1 [file plants-12-02177-s001.zip › plants-2411413-supplementary.pdf]

plants

MDPI

Supplementary Materials

Phytochemical Characterization of Saudi Mint and its Mediating Effect on the Production of Silver Nanoparticles and its Antimicrobial and Antioxidant Activities

Husam Qanash [1,*], Abdulrahman S. Bazaid [1], Naif K. Binsaleh [1], Bandar Alharbi [1], Nawaf Alshammari [2], Safa H. Qahl [3], Hayaa M. Alhuthali [4] and Abdullatiff A. Bagher [5]

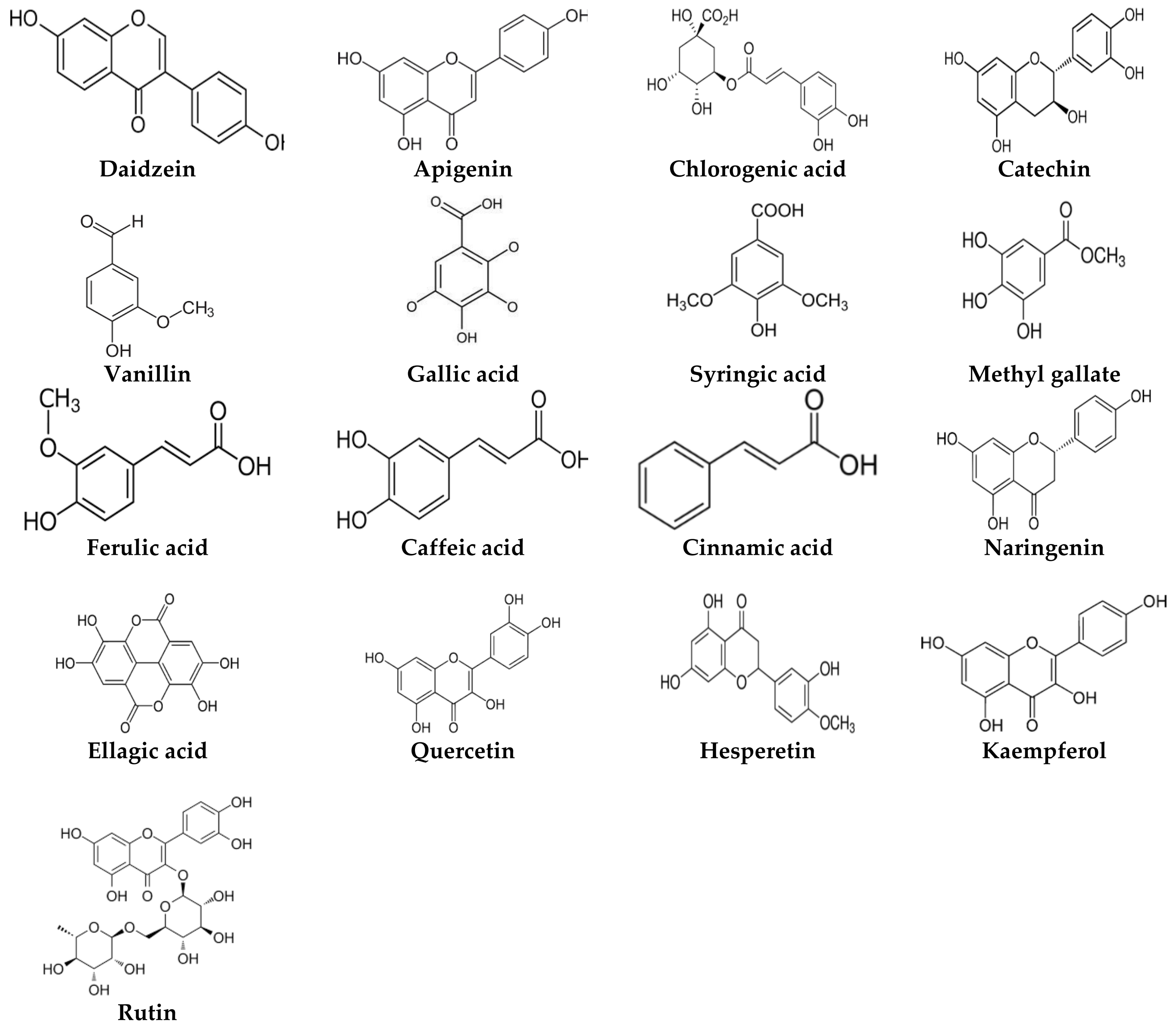

Figure S1. Chemical constructions of the identified phenolic and flavonoid in mint extract.
